# The Role of the Keap1/Nrf2 Pathway in the Cellular Response to Methylmercury

**DOI:** 10.1155/2013/848279

**Published:** 2013-06-26

**Authors:** Yoshito Kumagai, Hironori Kanda, Yasuhiro Shinkai, Takashi Toyama

**Affiliations:** ^1^Environmental Biology Section, Faculty of Medicine, University of Tsukuba, Tsukuba, Ibaraki 305-8575, Japan; ^2^Doctoral Program in Biomedical Sciences, Graduate School of Comprehensive Human Sciences, University of Tsukuba, Tsukuba, Ibaraki 305-8575, Japan

## Abstract

Methylmercury (MeHg) is an environmental electrophile that covalently modifies cellular proteins with reactive thiols, resulting in the formation of protein adducts. While such protein modifications, referred to as *S*-mercuration, are thought to be associated with the enzyme dysfunction and cellular damage caused by MeHg exposure, the current consensus is that (1) there is a cellular response to MeHg through the activation of NF-E2-related factor 2 (Nrf2) coupled to *S*-mercuration of its negative regulator, Kelch-like ECH-associated protein 1 (Keap1), and (2) the Keap1/Nrf2 pathway protects against MeHg toxicity. In this review, we introduce our findings and discuss the observations of other workers concerning the *S*-mercuration of cellular proteins by MeHg and the importance of the Keap1/Nrf2 pathway in protection against MeHg toxicity in cultured cells and mice.

## 1. Introduction

**Figure 1:**
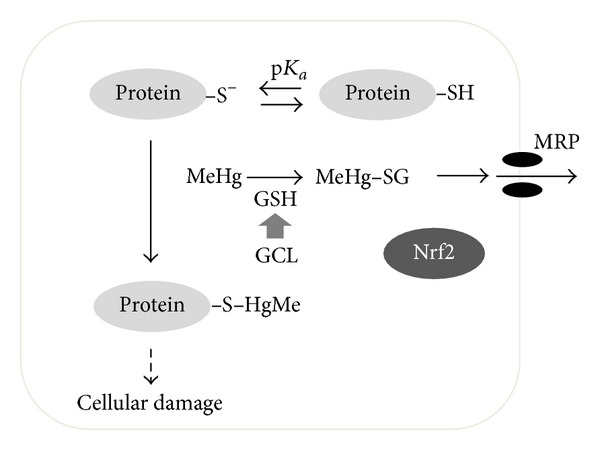
*S*-mercuration of cellular proteins by MeHg and the MeHg detoxification pathway.

 Methylmercury (MeHg) is strongly accumulated by fish and marine mammals and is found at the highest concentrations in large predatory species at the top of the aquatic food chain. MeHg readily penetrates the blood-brain barrier and affects the central nervous system. It is transferred into cells by passive diffusion and/or transport by the L-type large neutral amino acid transporter (as its L-cysteine complex) and reacts covalently with protein thiols because of its high affinity for nucleophilic groups, having a dissociation constant of 10^−15^ [1]. This covalent modification by MeHg is referred to as “*S*-mercuration.” Some MeHg, however, binds to glutathione (GSH) that is present in a variety of cell types at relatively high concentrations (>1 mM) to form a MeHg-GSH adduct that is transported into the extracellular space by multidrug resistance-associated proteins (MRPs) [2–4] (Figure 1). While the MeHg-mediated *S*-mercuration of cellular proteins is believed to be involved in the toxicological significance of MeHg [5], oxidative stress from the presence of MeHg in the body is also a critical factor associated with its toxicity [6]. Interestingly, antioxidant proteins such as heme oxygenase-1 (HO-1), glutamate-cysteine ligase (GCL; a rate-limiting enzyme for GSH synthesis), and MRPs are regulated by transcription factor NF-E2-related factor 2 (Nrf2) [7–12].

## 2. *S*-Mercuration of Proteins by MeHg

 Although there is little doubt that MeHg is able to covalently modify cellular proteins, and it is thought that a protein-MeHg complex is, at least partly, involved in MeHg toxicity (Figure 1), there have been only a limited number of studies in which the *in vivo* effects of MeHg have been investigated with regard to *S*-mercuration. This is probably because of the lack of appropriate methods for identifying *S*-mercuration. Here, we describe our efforts to investigate the effects of MeHg using chemical biology approaches (Table 1).

We have found that the subcutaneous administration of MeHg (10 mg/kg) to mice resulted in a time-dependent decrease in brain manganese-superoxide dismutase (Mn-SOD) activity, whereas MeHg exposure had no effect on cuprozinc-SOD (Cu,Zn-SOD) activity [13]. Although levels of mRNA and protein synthesis of Mn-SOD were unaffected by MeHg administration, MeHg caused a facile reduction in the activity, and a drastic decrease in the native form, of Mn-SOD (but not of Cu,Zn-SOD) with purified enzyme preparations. It was subsequently shown that the *S*-mercuration of Mn-SOD, through Cys196, by MeHg results in a decrease in the enzyme activity *in vivo* [14]. Therefore, the selective inhibition of Mn-SOD activity caused by *S*-mercuration could play a critical role in MeHg-induced neurotoxicity because SOD protects the cell against oxidative stress by scavenging superoxide.

We also found that the subcutaneous administration of MeHg (10 mg kg^−1^ day^−1^ for 8 days) to rats caused significant increases in nitric oxide synthase (NOS) activities in the cerebrum and cerebellum with time [15]. The increase in NOS activity seems to be caused by an increase in neuronal NOS (nNOS) protein levels, but not an increase in inducible NOS. In contrast to the *in vivo* observations, however, the addition of MeHg to a cerebellar enzyme preparation *in vitro* caused a concentration-dependent decrease in nNOS activity, suggesting that MeHg modifies NOS through reactive thiol groups, thereby affecting its catalytic activity *in vitro*. Experiments with arylmercury column chromatography have confirmed that this is possible [15]. A reasonable explanation for our observations is that the increase in nNOS proteins observed from MeHg exposure *in vivo* may be one of the initial responses to counteract the metal-induced negative effects on the central nervous system.

 In the body, MeHg accumulates to high concentrations in the liver [16, 17], but few molecular targets of MeHg have been identified in this tissue. We hypothesized that MeHg could interact with arginase I, an abundant Mn-binding protein in the liver, through covalent modification, resulting in alterations in not only its catalytic activity but also in hepatic Mn levels through *in vivo* MeHg exposure. After the subcutaneous administration of MeHg (10 mg kg^−1^ day^−1^ for 8 days) to rats, mercury levels in the liver were greater than those in the cerebrum or cerebellum [18]. A marked suppression of arginase I activity was also detected under these conditions. Using purified rat arginase I, we found that MeHg-induced *S*-mercuration of arginase I caused the protein to aggregate and substantial leakage of Mn ions from the arginase I active site. We speculate that the MeHg-mediated suppression of hepatic arginase I activity *in vivo* is, at least partly, attributable to the *S*-mercuration of this protein.

 In the course of the study, we coincidentally detected another protein, with a 42 kDa subunit molecule, from the same hepatic preparation that readily underwent *S*-mercuration and subsequent aggregation. Two-dimensional sodium dodecyl sulfate-polyacrylamide gel electrophoresis was used to separate the protein, and it was identified by peptide mass fingerprinting using matrix-assisted laser desorption and ionization time-of-flight mass spectrometry (MALDI-TOF/MS). This 42 kDa protein was identified as sorbitol dehydrogenase (SDH) [19]. Using recombinant rat SDH possessing 10 cysteine residues in a subunit [20], we found that MeHg was covalently bound to SDH through Cys44, Cys119, Cys129, and Cys164, resulting in the inhibition of its catalytic activity and the release of zinc ions, facilitating protein aggregation [19]. Subsequent mutation analysis confirmed that Cys44, which ligates the active site zinc atom [21], and Cys129 play a crucial role in the MeHg-mediated aggregation of SDH [19].

## 3. Cellular Response to MeHg via the Keap1/Nrf2 Pathway

 As mentioned above, MeHg reacts readily with cellular nucleophiles and also causes oxidative stress, implying that it may lead to decreased GSH levels in tissues. As a result, MeHg undergoes GSH conjugation and is excreted into the extracellular space through the action of MRPs [2–4]. However, several studies have indicated that MeHg exposure also upregulates GCL [22–28]. This suggests that there is an initial response to MeHg in the cells to compensate for decreased GSH levels and to repress oxidative cell damage. On the other hand, Dr. Yamamoto and his associates reported that transcription factor Nrf2 cooperatively regulates antioxidant proteins such as GCL and HO-1, phase-II xenobiotic detoxifying enzymes, and phase-III xenobiotic transporters such as MRPs [10, 29]. They subsequently found that Nrf2 is negatively regulated by Kelch-like ECH-associated protein 1 (Keap1) [30]. Interestingly, Keap1 has 27 and 25 cysteine residues in humans and mice, respectively [31, 32], and some thiols (e.g., Cys151, Cys273, and Cys288) that have been established as being highly reactive [31–34], so the “cysteine code,” which defines the preferential target cysteine(s) and distinct biological effects, was proposed [35–37]. These observations led us to assume that MeHg could modify Keap1 through *S*-mercuration and so activate the Nrf2-regulated gene expression of GCL, HO-1, and MRPs.

**Figure 2:**
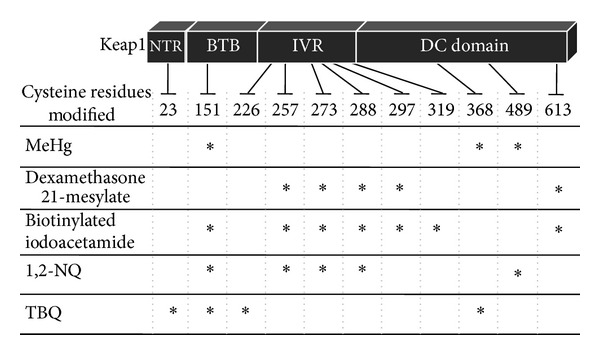
Covalent modification of Keap1 cysteine residues by electrophiles. The BTB, IVR, and DC domains are essential for the formation of homodimers, associated with cullin 3 and the binding of Nrf2. 1,2-NQ: 1,2-naphthoquinone; TBQ: *tert*-butyl-1,4-benzoquinone.

**Figure 3:**
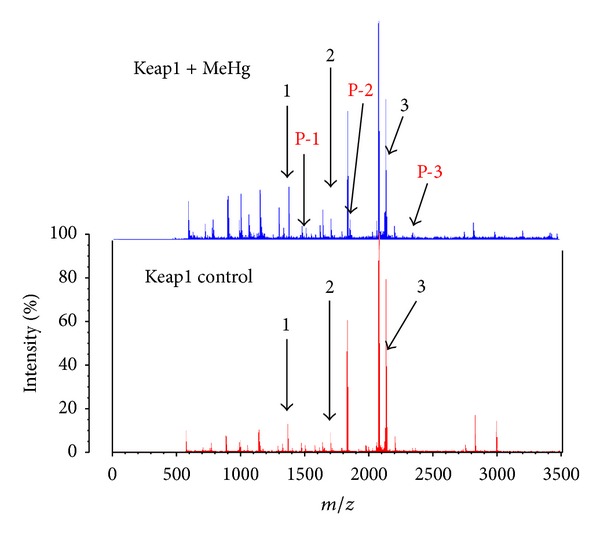
MALDI-TOF/MS analysis of Keap1 reacted with MeHg. Recombinant Keap1 protein (0.25 *μ*g/*μ*L) was incubated for 30 min at 37°C with or without MeHg (10 *μ*M). After the reaction, the Keap1 proteins were trypsinized and subjected to MALDI-TOF/MS analysis. Compared with the calculated mass of the unmodified peptides, the modified peptides P-1 to P-3 showed increased masses of 214 or 216 Da, corresponding to the addition of a single equivalent of MeHg.

Figure 2 shows a summary of the Keap1 modification sites, consisting of NTR (N-terminal region), BTB (Board complex, Tramtrack, and Bric-à-brac), IVR (intervening region), and DC (DGR/CTR: double glycine repeat/C-terminal region) domains that can be modified by a variety of electrophiles [31, 33, 38, 39]. Among them, Cys151, Cys273, and Cys288 are well established as being essential for regulating Nrf2 function [31, 32, 40, 41], although other cysteine residues (e.g., Cys257, Cys297, and Cys613) in Keap1 can also be modified by dexamethasone 21-mesylate, biotinylated iodoacetamide, and 1,2-napthoquinone [31, 33, 38]. Our MALDI-TOF/MS analysis revealed that Keap1 undergoes *S*-mercuration by MeHg at three cysteine residues, Cys151, Cys368, and Cys489 (see Figure 3 and Table 2), suggesting that the *S*-mercuration of Cys151 in Keap1 potentially causes a structural alteration, activating Nrf2, while Cys368 and Cys489 are also targets for 1,2-naphthoquinone and *tert*-butyl-1,4-benzoquinone [38, 39].

**Figure 4:**
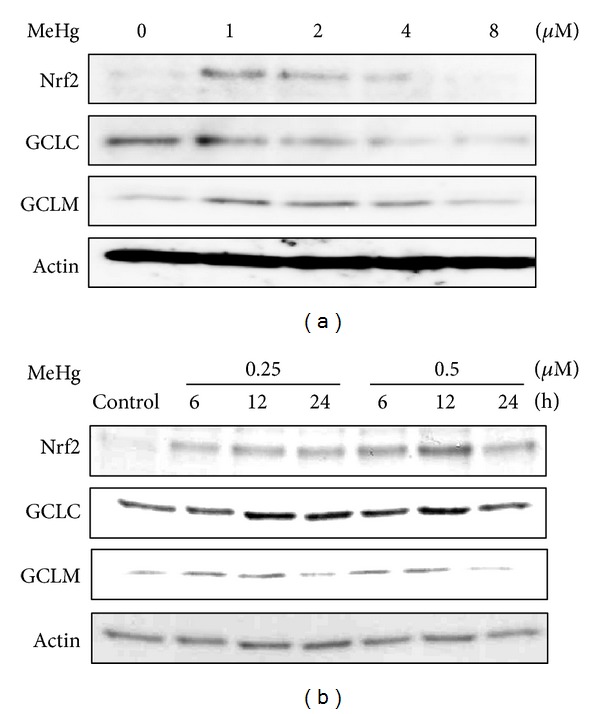
MeHg activates Nrf2 in SH-SY5Y cells. (a) Cells were exposed to MeHg (1, 2, 4, or 8 *μ*M) for 6 h, and the total cell lysates (20 *μ*g) were subjected to western blotting with the antibodies indicated. (b) Cells were exposed to MeHg (0.25 or 0.5 *μ*M) for 6, 12, or 24 h, and the total cell lysates (20 *μ*g) were subjected to western blotting with the antibodies indicated. Reprinted from Toyama et al. [42] with the permission of Biochemical Biophysical Research Communications.

As expected, MeHg activated Nrf2 in SH-SY5Y cells in a concentration- and time-dependent manner and upregulated the downstream proteins such as the GCL catalytic subunit (GCLC) and the GCL modifier subunit (GCLM) (Figure 4) [42]. The same observation was seen in primary mouse hepatocytes and HepG2 cells (data not shown). Other researchers also reported that MeHg led to Nrf2 activation, its nuclear accumulation, and upregulation of Nrf2 downstream antioxidant genes such as HO-1 in a variety of cell types [43–45]. Interestingly, Ni et al. demonstrated different response kinetics in astrocytes and microglia upon MeHg treatment [46]. They concluded that these unique sensitivities appear to be dependent on the cellular thiol status of the particular cell type. This viewpoint is consistent with the evidence that reactive thiols of Keap1 undergo *S*-mercuration, resulting in Nrf2 activation. Pretreatment with Trolox, an antioxidant, blocked MeHg-mediated oxidative stress, determined by intracellular reactive oxygen species levels, to a significant degree but did not affect Nrf2 activation during the exposure of SH-SY5Y cells to MeHg (Toyama et al., unpublished data). We therefore speculate that *S*-mercuration, rather than oxidative stress, is involved in the MeHg-dependent activation of Nrf2.

## 4. Protection against MeHg Toxicity through the Keap1/Nrf2 Pathway

**Figure 5:**
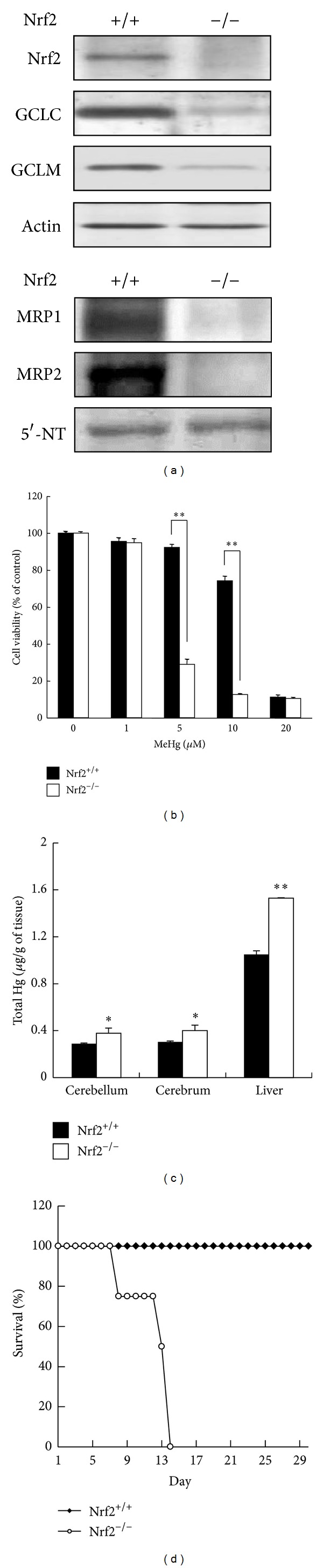
Nrf2 confers protection against MeHg toxicity. (a) Total cell lysates (upper) or crude membrane fractions (lower) from primary hepatocytes from Nrf2^+/+^ or Nrf2^−/−^ mice were subjected to western blotting with the antibodies indicated. (b) Primary hepatocytes from Nrf2^+/+^ or Nrf2^−/−^ mice were exposed to MeHg (1, 5, 10, and 20 *μ*M) for 24 h, and then the MTT assay was performed. Each value represents the mean ± SE of three independent experiments. (c) Nrf2^+/+^ or Nrf2^−/−^ mice were orally administrated MeHg (1 mg/kg). After 48 h, the total mercury contents of the cerebellum, cerebrum, and liver were determined by atomic absorption mercury detection. Each value represents the mean ± SE of five independent experiments. (d) Nrf2^+/+^ or Nrf2^−/−^ mice were orally administered MeHg (5 mg kg^−1^ day^−1^) for 12 days (*n* = 4) and mortality was recorded. *P < 0.05 and **P < 0.01 for the Nrf2^−/−^ mice compared to the Nrf2^+/+^ mice. Partially reprinted from Toyama et al. [42, 53] with the permission of Biochemical Biophysical Research Communications and Environmental Health Perspectives.

**Figure 6:**
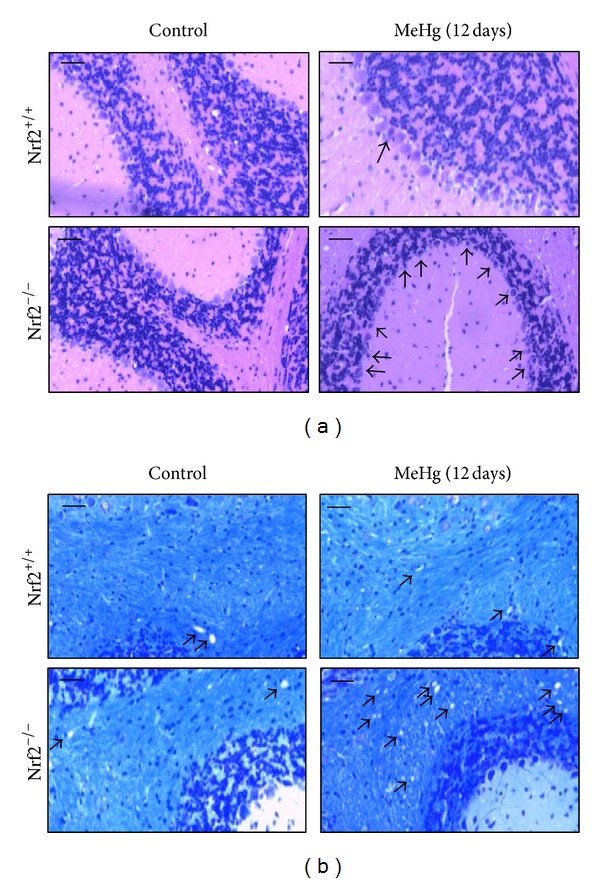
Pathological observations of brains from Nrf2^+/+^ or Nrf2^−/−^ mice given MeHg. Nrf2^+/+^ or Nrf2^−/−^ mice were orally administrated MeHg (5 mg kg^−1^ day^−1^) for 12 days, and pathophysiological changes in the cerebellum were observed. Bar = 50 *μ*m. (a) Photographs of the cerebellar granule cell layer (HE stain). Arrows indicate the MeHg-induced degeneration of Purkinje cells. (b) Photographs of the cerebellum medulla (KB stain). Arrows indicate the MeHg-induced vacuolar degeneration.

 Several lines of evidence indicate that Nrf2-deficient mice are susceptible to the toxicity of a variety of chemicals, including acetaminophen, 7,12-dimethylbenz[*a*]anthracene, kainic acid, dextran sulfate sodium, and benzo[*a*]pyrene [47–52]. To examine the role of Nrf2 in protecting against MeHg, we used primary hepatocytes from Nrf2^+/+^ or Nrf2^−/−^ mice. As shown in Figure 5(a), steady-state levels of Nrf2 downstream proteins, such as GCLC, GCLM, MRP1 [42], and MRP2 [42], were drastically decreased in Nrf2^−/−^ cells. Under these condition, the Nrf2^−/−^ cells were highly sensitive to MeHg compared with the Nrf2^+/+^ cells (Figure 5(b)) [42]. In agreement with these observations, Ni et al. demonstrated that Nrf2 knockdown by the small hairpin RNA approach reduced the upregulation of its downstream genes such as HO-1 in primary astrocytes and microglia and decreased viability for both cells [45, 46]. The accumulation of mercury in the cerebellum, cerebrum, and liver after oral MeHg administration was significantly higher in the Nrf2^−/−^ mice than in the Nrf2^+/+^ mice (Figure 5(c)) [53] because detoxifying enzymes associated with MeHg excretion were relatively low in the Nrf2^−/−^ mice. Consistent with this result, the Nrf2^−/−^ mice were also highly susceptible to MeHg *in vivo* (Figure 5(d)). MeHg-induced neuropathological changes in the cerebellum were evaluated in the Nrf2^+/+^ and Nrf2^−/−^ mice (Figure 6), and the degeneration of Purkinje cells (Figure 6(a)) and vacuolar degeneration of the medulla (Figure 6(b)) were observed in the cerebellum of MeHg-treated mice. Nrf2 deletion clearly increased these types of MeHg-induced degeneration. These results suggest that Nrf2 is a crucial transcription factor for protecting against MeHg toxicity *in vitro* and *in vivo*.

## 5. Conclusions

**Figure 7:**
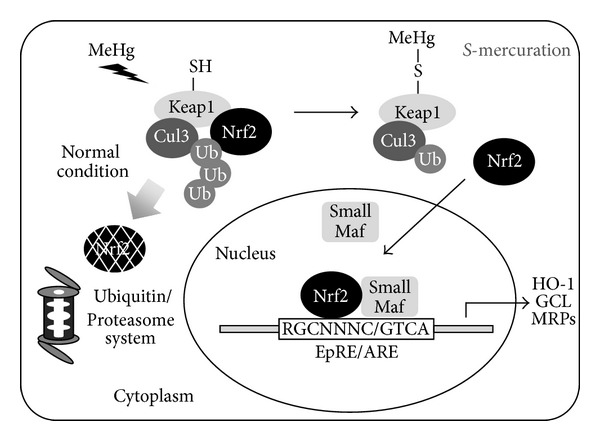
Cellular protective response to MeHg through the Keap1-Nrf2 system.

 We found that MeHg *S*-mercuration reactions on Mn-SOD and arginase I cause dysfunction in the activities of these catalysts and, therefore, possibly cause oxidative stress in mouse brain and the release of Mn from the active site, leading to a decrease in hepatic Mn levels (seen in rats). The evidence showed that MeHg also *S*-mercurates Keap1 at its reactive thiols, including Cys151, resulting in Nrf2 activation in a variety of cell types, and thereby upregulation of the downstream gene products such as antioxidant proteins, phase II xenobiotic-metabolizing enzymes, and phase III transporters (as shown in Figure 7). Because these Nrf2-regulated gene products contribute to the blocking of oxidative stress and the facilitation of the detoxification and excretion of MeHg, the findings indicate that the Keap1/Nrf2 system plays a critical role not only in the cellular response to MeHg, but also in the suppression of MeHg toxicity *in vitro* and *in vivo*.

## Figures and Tables

**Table 1:** Molecular targets of MeHg.

Target protein	*In vivo* effect	References
Mn-SOD	Reduction of catalytic activity in mouse brain	Shinyashiki et al. [13]
nNOS	Induction of NOS in rat brain	Shinyashiki et al. [15]
Arginase I	Reduction of activity in rat liver Decreased Mn levels in tissues	Kanda et al. [18]
SDH	Not defined	Kanda et al. [19]

**Table 2:** MeHg modification sites in Keap1.

Peak no.	Position	Peptide sequence	Calculated mass	Observed mass
1	484–494	LNSAECYYPER	1345.5	1345.1
2	363–380	SGLAGCVVGGLLYAVGGR	1649.9	1649.6
3	151–169	CVLHVMNGAVMYQIDSVVR	2135.6	2135.1
P-1	484–494 +MeHg	LNSAE**C**YYPER +MeHg (214)	1560.5	1559.6
P-2	363–380 +MeHg	SGLAG**C**VVGGLLYAVGGR +MeHg (216)	1863.8	1864.8
P-3	151–169 +MeHg	**C**VLHVMNGAVMYQIDSVVR +MeHg (214)	2350.6	2349.5
